# Regulatory Role of Jasmonate Signaling in Dark-, Drought-, and Salt-Induced Leaf Senescence

**DOI:** 10.3390/ijms27041725

**Published:** 2026-02-11

**Authors:** Marzena Kurowska, Boris Lazarević

**Affiliations:** 1 Institute of Biology, Biotechnology and Environmental Protection, Faculty of Natural Sciences, University of Silesia in Katowice, Jagiellońska 28, 40-032 Katowice, Poland; 2 Faculty of Agriculture, University of Zagreb, 10000 Zagreb, Croatia; blazarevic@agr.hr; 3 Centre of Excellence for Biodiversity and Molecular Plant Breeding (CroP-BioDiv), 10000 Zagreb, Croatia

**Keywords:** jasmonate, OPDA, drought, salinity, senescence, chlorophyll degradation, mutants

## Abstract

Drought and salinity are among the most severe abiotic stresses limiting global agricultural productivity, and their frequency and intensity are expected to increase under ongoing climate change. Concurrently, the growing human population necessitates the development of crop varieties that combine high yield with enhanced stress tolerance. Jasmonates, including jasmonic acid (JA) and its derivatives, play pivotal roles in plant responses to abiotic stresses and are widely regarded as stress hormones. The bioactive conjugate jasmonoyl-isoleucine (JA-Ile) acts as a ligand for the coronatine insensitive 1 (COI1) receptor. Jasmonates regulate essential physiological processes, including leaf senescence, secondary metabolism, and nutrient homeostasis, which collectively contribute to plant adaptation to drought and salinity stress. In this review, we summarize recent advances in understanding the role of jasmonate signaling in plant responses to drought and salinity, with particular emphasis on the initiation and progression of leaf senescence and chlorophyll degradation pathways. By integrating genetic, biochemical, and physiological evidence, we discuss how targeted modulation of jasmonate levels and signaling components may be exploited to breed or engineer crops with improved tolerance to water-deficit and saline conditions.

## 1. Introduction

Jasmonates are a class of oxylipin-derived plant hormones that regulate numerous physiological processes during plant growth and development, as well as responses to environmental stress. Chemically, they are cyclic derivatives of unsaturated fatty acids. JA, a twelve-carbon cyclopentanone derivative, is the core compound of this group, and the term jasmonates encompasses JA and its derivatives, including methyl jasmonate (MeJA), one of the most extensively studied and earliest identified members of this hormone family. JA was initially isolated as a plant growth inhibitor from culture filtrates of the fungus *Lasiodiplodia theobromae* [1], whereas MeJA was first identified in the essential oils of *Jasminum grandiflorum* [2] and *Rosmarinus officinalis* [3]. Jasmonates are ubiquitously distributed in higher plants and can be detected in stems, roots, tubers, leaves, fruits, and floral pollen [4].

Jasmonates are often referred to as stress hormones due to their pivotal role in plant responses to both biotic and abiotic stress. They participate in defense mechanisms against herbivores, nematodes, and pathogens, as well as in responses to drought, salinity, osmotic stress, heavy metals, micronutrient toxicity, ultraviolet radiation, extreme temperatures, elevated CO_2_, light stress, and ozone exposure. Additionally, jasmonates mediate responses to mechanical stress, including tissue damage caused by herbivory or physical injury [4,5,6]. Through these functions, jasmonates enable plants to balance growth and defense strategies, thereby influencing survival, crop quality, and yield. Beyond stress adaptation, jasmonates regulate a wide range of developmental processes, including leaf senescence, tuber formation, seed germination, root growth inhibition, flower and reproductive organ development, lateral root formation, stamen and trichome development, cell cycle regulation, fruit ripening, stomatal movement, nutrient uptake (nitrogen and phosphorus), glucose transport, gravitropism, light signaling, flowering repression, fertility, and microbial symbioses [5,6,7]. They also play a crucial role in the biosynthesis of secondary metabolites such as alkaloids, taxol, and anthocyanins, and are involved in chlorophyll degradation and tumor suppression, highlighting potential applications in pharmaceutical production [5,8,9]. JA is a major component of floral volatiles and has commercial value in the perfume industry, as well as in the production of toiletries, chewing gum, and cigarettes [10].

Leaf senescence represents a highly coordinated developmental program that enables the remobilization of nutrients from aging leaves to growing or reproductive tissues [11,12]. Under natural conditions, senescence progression is tightly regulated by endogenous developmental cues and environmental signals [13,14]. However, under abiotic stress conditions such as drought and salinity, senescence can be prematurely induced or accelerated, resulting in reduced photosynthetic capacity and yield losses [15,16]. Because jasmonates are closely associated with both stress responses and senescence-related processes, they have been widely implicated in the regulation of stress-induced leaf senescence.

Despite this broad functional spectrum, the contribution of jasmonates to leaf senescence is strongly influenced by developmental stage, environmental conditions, and the experimental or physiological context in which senescence is studied [17,18]. Much of our current understanding of jasmonate-induced senescence has been derived from controlled experimental systems, including dark-induced leaf senescence and exogenous jasmonate treatments, which have been instrumental in elucidating core signaling components. Under abiotic stress conditions, jasmonate-induced responses frequently operate in coordination with other stress signaling pathways and hormonal networks, collectively shaping senescence progression and plant performance under field-relevant conditions [19,20]. In addition, recent studies have highlighted context-dependent jasmonate signaling modes, including both COI1-dependent and COI1-independent pathways [7,21].

Drought and salinity are among the most critical abiotic stresses affecting crop yield and performance, and ongoing climate change is expected to exacerbate their impact. Recent assessments indicate an increased frequency of temperature extremes, heatwaves, and water-related stress events across major agricultural regions, with negative consequences for crop productivity [22]. Although global agricultural productivity has improved over recent decades, climate change has moderated these gains, underscoring the need for improved understanding of stress-response mechanisms and their translation into breeding and crop management strategies [22].

The objective of this review is to summarize and integrate current knowledge on jasmonate signaling in leaf senescence induced by darkness, drought, and salinity. Particular emphasis is placed on comparing insights obtained from different experimental systems and stress contexts, with the aim of clarifying the contribution of jasmonates to stress-induced senescence and their relevance for plant performance under adverse environmental conditions.

## 2. Principle of Jasmonate Biosynthesis and Signaling Pathways

**Figure 1:**
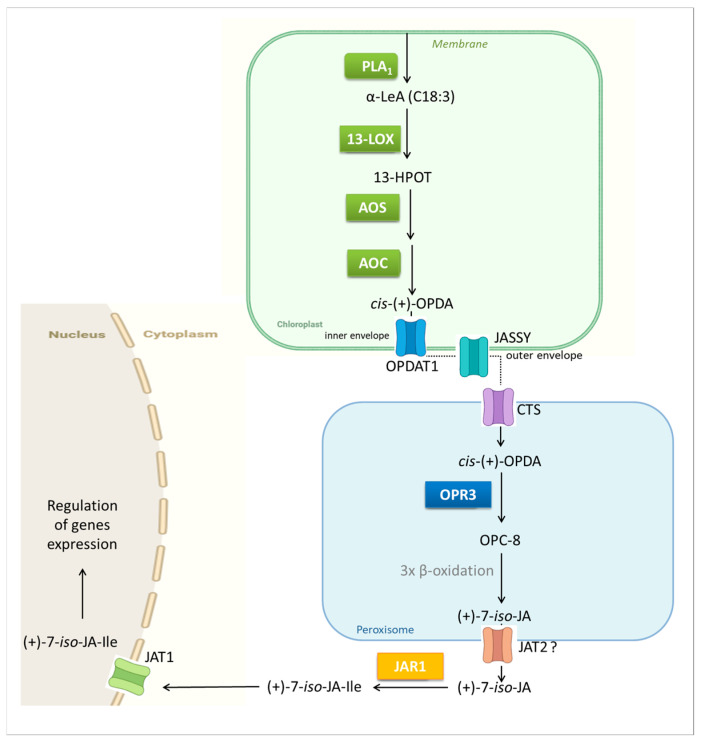
Key steps of JA biosynthesis in plants and intracellular transport of intermediates. Created in BioRender. Kurowska, M. (2026) https://BioRender.com/u2lr2xt, accessed on 10 January 2026. Based on Wasternack et al. [5] and Kamali et al. [34].

Jasmonates, including JA and MeJA, serve as precursors or derivatives of the bioactive jasmonate form. In plants, the bioactive form of jasmonates has been identified as a JA-Ile, which functions as a ligand for the JA receptor [23]. The biosynthesis of JA in plant cells is a tightly regulated, multi-step process that occurs across several cellular compartments. It begins in the chloroplasts, continues in the peroxisomes, and is completed in the cytoplasm [24]. This complexity reflects the diverse physiological roles of jasmonates and the need for precise control over their production. In most plants, JA and its derivatives are synthesized from α-linolenic acid (C18:3; α-LeA), which is released from chloroplast membranes by phospholipases such as defective in anther dehiscence 1 (DAD1) or dongle (DGL), members of the phospholipase A_1_ (PLA_1_) family. In some species, JA can also be produced from the 16-carbon substrate 7Z,10Z,13Z-hexadecatriene acid (C16:3) [24]. Both C18:3 and C16:3 fatty acids are converted through the action of lipases belonging to the 13-lipoxygenase (13-LOX) family. In *Arabidopsis*, four such enzymes have been identified: LOX2, LOX3, LOX4, and LOX6. Subsequently, allene oxide synthase (AOS) and allene oxide cyclase (AOC) catalyze sequential steps in the pathway, converting C18:3 to 12-oxophytodienoic acid (OPDA) and C16:3 to dinor-oxophytodienoic acid (dnOPDA). These intermediates are synthesized in the chloroplasts and transported to the peroxisomes, where they are reduced by 12-oxophytodienoic acid reductase (OPR3) and subsequently undergo three rounds of β-oxidation mediated by acetyl-CoA oxidase (ACX). These transformations yield (+)-7-iso-jasmonic acid, which can epimerize to JA. After transport to the cytoplasm, JA can be conjugated with isoleucine to form the biologically active JA-Ile (Figure 1).

In recent years, significant progress has been made in elucidating the transport mechanisms of intermediates involved in jasmonate biosynthesis. Key chloroplast transport proteins have been identified, including OPDA transporter 1 (OPDAT1), located in the inner envelope, and jasmonate-associated secretory system (JASSY), which is localized in the outer envelope membrane [25,26]. In addition, the ABC transporter comatose (CTS), located in the peroxisomal membrane, facilitates substrate import, and jasmonate transporter 2 (JAT2), a candidate for peroxisomal export, has also been identified [27,28,29,30,31]. Finally, JAT1, which is responsible for transporting JA-Ile into the nucleus, represents one component of the known pathways of jasmonate intracellular movement [30,32,33] (Figure 1).

**Figure 2:**
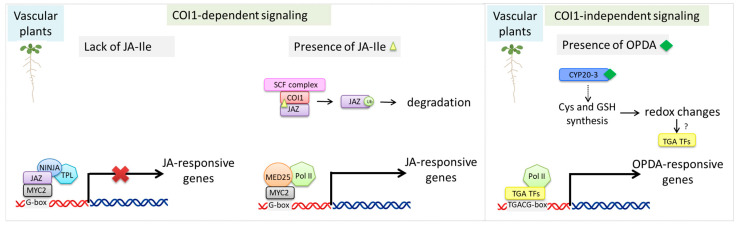
Principle of jasmonate signal transduction in vascular plants via COI1-dependent and COI1-independent signaling pathways. In the COI1-dependent pathway, jasmonic acid (JA) is conjugated with isoleucine by JAR1 to form the bioactive jasmonate JA-Ile, which is perceived by the SCF^COI1 complex. Binding of JA-Ile promotes the interaction between COI1 and JAZ repressors, leading to JAZ ubiquitination and degradation via the 26S proteasome. This releases transcription factors (TFs), enabling the recruitment of mediator complex subunit 25 (MED25) and RNA polymerase II (Pol II) and subsequent activation of jasmonate-responsive gene expression. In the COI1-independent pathway, OPDA signaling involves redox-dependent mechanisms associated with cyclophilin 20-3 (CYP20-3), cysteine (Cys), and glutathione (GSH), contributing to transcriptional regulation independently of COI1. Created in BioRender. Kurowska, M. (2026) https://BioRender.com/7oh8l9d, accessed on 10 January 2026. Based on Park et al. [38] and Wasternack et al. [5].

The JA signal is perceived in the cell nucleus and involves the ubiquitin–proteasome system, which also mediates signal transduction for other plant hormones. This system includes the Skp1/Cullin/F-box (SCF) complex, which functions as an E3 ubiquitin ligase, and the JA receptor COI1, which serves as the F-box component of this complex (Figure 2) [35,36]. Jasmonate ZIM-domain (JAZ) proteins, which act as negative regulators of JA-responsive gene expression, are the primary targets of SCF/COI1-mediated proteolytic degradation [37]. Under stress conditions, endogenous JA-Ile levels rise, triggering the degradation of JAZ repressors. This degradation releases transcription factors such as MYC2, allowing them to bind *cis*-regulatory elements in promoters of JA-responsive genes and activate their expression (Figure 2). Conversely, when JA-Ile levels are low, JAZ proteins inhibit MYC2 activity by recruiting topless (TPL) co-repressors and TPL-related proteins through interactions with the novel interactor of JAZ (NINJA) adapter proteins. This mechanism effectively blocks the transcription of JA-induced genes, maintaining the pathway in an inactive state (Figure 2).

OPDA, a key precursor in jasmonate biosynthesis, has also been reported to participate in signaling processes that can operate independently of the canonical COI1-dependent jasmonate pathway (Figure 2) [38,39,40,41,42,43,44]. Significant accumulation of OPDA and dnOPDA has been observed across various plant species [45]. OPDA is closely linked to responses to environmental stress, with its accumulation increasing during drought and being associated with stomatal responses that differ from those mediated by JA or MeJA [46,47]. In *Arabidopsis*, OPDA has been shown to influence stomatal closure more effectively than MeJA under drought conditions [46] and, unlike JA, to inhibit light-triggered stomatal opening [47], underscoring the distinct functional roles of individual oxylipins. In addition to its roles in stress-associated stomatal regulation, OPDA has been implicated in developmental processes, including the differentiation of leaf primordia, seed germination, and embryo development [48,49,50,51].

CYP20-3, also known as ROC4, has been proposed as an OPDA effector involved in redox regulation (Figure 2) [38,52]. OPDA binding promotes the formation of the cysteine synthase complex (CSC), composed of serine acetyltransferase 1 (SAT1) and O-acetylserine(thiol)lyase B (OASTL-B), which enhances Cys and subsequently GSH production. These redox changes activate TGACG-binding (TGA) transcription factors that regulate OPDA-responsive genes [53]. OPDA can also covalently modify cysteinyl residues in proteins via OPDAylation, directly altering protein function [7,54,55].

Although evidence supporting OPDA-associated signaling responses is steadily growing, the underlying mechanisms remain incompletely characterized and appear to represent a regulatory layer that is at least partially distinct from canonical jasmonate pathways. Importantly, current evidence does not yet allow a clear distinction between OPDA acting as a primary regulatory signal and OPDA accumulation reflecting broader oxylipin metabolism during stress-associated tissue remodeling. Clarifying these relationships will be essential for accurately interpreting OPDA function in complex physiological contexts such as stress-induced leaf senescence.

Elucidating OPDA-specific pathways may also uncover novel regulatory elements that can be leveraged to enhance plant resilience, optimize resource use, and improve tolerance to both biotic and abiotic stresses.

## 3. Role of Jasmonate Signaling in Leaf Senescence Under Drought and Salt Stress

### 3.1. Leaf Senescence as a Developmental and Stress-Induced Process

**Figure 3:**
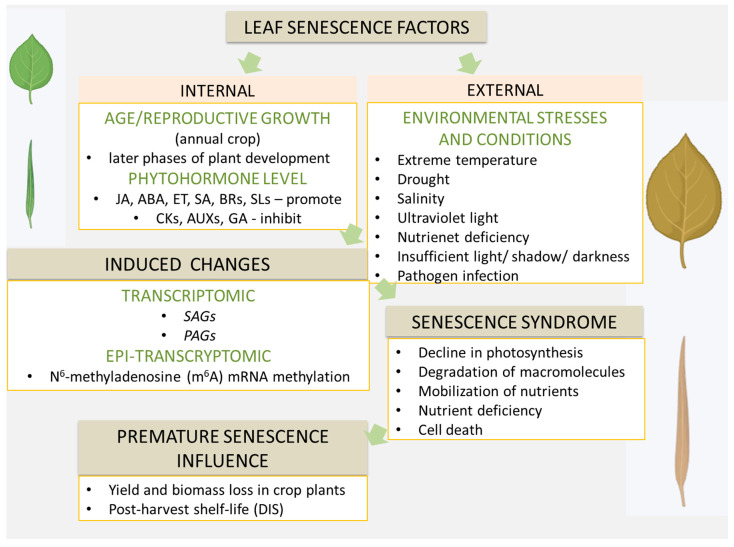
Examples of different factors which induced leaf senescence, associated syndromes, transcriptomic and epi-transcriptomic changes.

Leaf senescence is a genetically programmed process influenced by environmental factors and occurs during the later stages of plant development [56]. It is generally categorized into three types: natural (age-dependent), starvation- or dark-induced, and stress-induced senescence [53]. Senescence is primarily regulated by endogenous developmental cues, such as plant age and reproductive growth in annual crops [57,58,59]. Phytohormones play a central role in this regulation: JA, abscisic acid (ABA), ethylene (ET), salicylic acid (SA), brassinosteroids (BRs), and strigolactones (SLs) promote senescence, whereas cytokinins (CKs), auxins (AUXs), and gibberellins (GAs) act as inhibitors [14,59,60,61,62,63,64,65,66,67,68,69,70,71] (Figure 3). Environmental stresses, including extreme temperatures, drought, salinity, ultraviolet radiation, nutrient deficiencies, low light, and pathogen attack, can accelerate senescence and induce its premature onset [72,73,74,75,76,77,78,79] (Figure 3). This process is accompanied by extensive transcriptomic and epi-transcriptomic reprogramming [80,81,82,83,84,85,86,87,88,89,90,91]. Key transcription factor families involved include WRKY, NAC (No Apical Meristem [NAM], *Arabidopsis* Transcription Activation Factor [ATAF], and Cup-shaped Cotyledon [CUC]), basic Helix-Loop-Helix (bHLH), APETALA2/Ethylene Response Factor (AP2/ERF), MYB, and DNA-binding with One Finger (DOF) proteins [92,93,94,95,96,97,98,99,100,101,102,103,104,105,106,107] (Figure 3). Among these, the NAC transcription factor Oresara1 (ORE1/ANAC092) and specific WRKY proteins act as central regulators [108,109,110,111] (Figure 3). Epi-transcriptomic modifications, particularly N^6^-methyladenosine (m^6^A) mRNA methylation, also influence leaf senescence by destabilizing senescence-related transcripts and preventing premature aging [112]. The molecular and biochemical changes associated with senescence collectively define the senescence syndrome, characterized by reduced photosynthetic capacity, cellular damage, macromolecule degradation, nutrient remobilization from older to developing organs, and eventual nutrient depletion [113,114,115,116]. In its terminal stage, senescence may culminate in programmed cell death [115].

### 3.2. Molecular and Physiological Hallmarks of Leaf Senescence

In crops, premature senescence has major agronomic and economic consequences, including reduced yield and biomass, as well as altered post-harvest shelf life [15,57,79,117] (Figure 3). Mature leaves are the main sites of carbon (C) assimilation through photosynthesis and serve as sources of C, which is exported to sink tissues such as developing seeds. During senescence, leaves transition from C sources to nitrogen (N) sources, with N derived from protein degradation being exported to young, expanding leaves acting as sinks at this stage [87]. The balance between C and N during these source–sink transitions is critical not only for senescence induction but also for shaping agronomic traits, including carbohydrate/nitrogen (C/N) use efficiency and yield [118,119,120,121].

**Figure 4:**
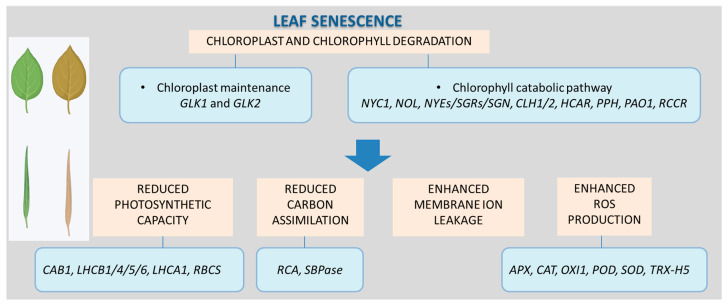
Leaf senescence symptoms and examples of genes whose expression is modulated during this process.

Chlorophyll (Chl) degradation and chloroplast disassembly are key hallmarks of leaf senescence, visually manifested as changes in leaf coloration [57,121,122,123,124,125]. Chl *b* degradation is initiated by reductase enzymes such as nonyellow coloring 1 (NYC1) and NYC1-like (NOL) [126], whereas Chl *a* catabolism is triggered by nonyellowing/stay-green (NYE/SGR/SGN) enzymes [127,128]. Other enzymes involved in Chl breakdown include chlorophyllase (CLH1/2), 7-hydroxymethyl chlorophyll *a* reductase (HCAR), pheophorbide *a* oxygenase 1 (PAO1), pheophytinase (PPH), and red chlorophyll catabolite reductase (RCCR) [14,122,129,130] (Figure 4).

During chloroplast disassembly, the expression of Golden2-like transcription factors (GLK1 and GLK2), which are master regulators of chloroplast maintenance, is repressed [131]. These processes collectively result in reduced photochemical efficiency [112,125,132], increased membrane ion leakage [58,112,133], decreased carbon assimilation [133], and elevated production of reactive oxygen species (ROS) [99,134] (Figure 4).

Gene expression changes underlie these phenotypic alterations. Two main classes are particularly important: *Senescence-Associated Genes* (*SAGs*) and *Photosynthesis-Associated Genes* (*PAGs*) [135]. Among the most studied photosynthesis-related genes are *Ribulose Bisphosphate Carboxylase Small Chain* (*RBCS*), *Chlorophyll a/b Binding Protein 1* (*CAB1*), *Light-Harvesting Complex of PSII Subunits 1/4/5/6* (*LHCB1/4/5/6*, also referred to as ‘antenna’ proteins), and *PSI Light-Harvesting Complex Gene 1* (*LHCA1*) [84,99,136,137]. Reduced carbon assimilation during senescence correlates with lower expression of Calvin cycle enzymes, including rubisco activase (RCA) and sedoheptulose-1,7-bisphosphatase (SBPase) [138,139].

Conversely, enhanced ROS production is linked to the upregulation of genes encoding antioxidant defense enzymes, such as *Ascorbate Peroxidase* (*APX*), *Catalase* (*CAT*), *Oxidative Signal Inducible 1* (*OXI1*), *Peroxidase* (*POD*), *Superoxide Dismutase* (*SOD*), and *Thioredoxin H5* (*TRX-H5*) [99] (Figure 4).

### 3.3. Jasmonate-Induced Regulation of Leaf Senescence

Jasmonate signaling is a key molecular mechanism regulating leaf senescence [60]. The first experimental evidence was provided by Ueda and Kato [140], who identified MeJA, the methyl ester of JA, as a senescence-promoting compound in oat (*Avena sativa* L.) derived from wormwood (*Artemisia absinthium* L.). Since then, MeJA has been widely used to induce leaf senescence in both model and crop species, including apple (*Malus domestica*), *Arabidopsis thaliana*, barley (*Hordeum vulgare* L.), Chinese flowering cabbage (*Brassica rapa*), rice (*Oryza sativa*), tobacco (*Nicotiana tabacum* L.), tomato (*Solanum lycopersicum*), and wheat (*Triticum aestivum* L.) [85,98,99,104,112,125,132,133,141,142,143,144]. MeJA is widely used as an experimental tool to activate jasmonate responses. Because evidence for jasmonate involvement in senescence comes from both induction assays (MeJA; dark-induced leaf senescence, DILS) and stress-induced senescence (drought, salinity), the following sections separate findings from standardized laboratory systems from observations made under progressive abiotic stress.

### 3.4. Interpreting Jasmonate Accumulation During Leaf Senescence

Exogenous MeJA accelerates senescence in both attached leaves (via spraying) and detached leaves (by floating on hormone solutions). MeJA treatment is often combined with dark incubation (DILS; Table 1), with these factors acting synergistically to promote rapid and uniform senescence. Senescence progression is typically characterized by increased JA accumulation, chlorophyll degradation, necrosis, changes in chloroplast morphology (from lenticular to spherical), thylakoid swelling, plastoglobuli formation, reduced photosynthetic efficiency, enhanced membrane ion leakage, and elevated ROS production which can further reinforce senescence-associated cellular damage [84,145] (Table 1). Photosynthetic performance is commonly assessed via parameters such as electron transport rate (ETR), maximum quantum efficiency of PSII (Fv/Fm), non-photochemical quenching (NPQ), relative fluorescence decline (RFD_Lss), and photochemical quenching (qP). Oxidative stress is measured by malondialdehyde (MDA) levels as an indicator of lipid peroxidation, 3,3′-diaminobenzidine (DAB) staining for H_2_O_2_ accumulation, and evaluation of membrane integrity. These changes are accompanied by increased activities of antioxidant enzymes, including SOD, CAT, APX, and POD (Figure 4). However, the most visible indicator of premature senescence remains leaf yellowing due to chlorophyll breakdown. While these physiological and molecular changes are reproducibly observed in MeJA- and DILS-based assays, their interpretation in terms of causal jasmonate function during senescence initiation remains challenging.

### 3.5. Causality Versus Correlation in Jasmonate-Induced Leaf Senescence

Genetic studies have provided key insights into the molecular mechanisms underlying JA-induced leaf senescence. Treatment with exogenous MeJA under dark conditions induces the expression of *SAGs* (Table 1), a phenomenon observed in several species, including apple (*MdSAG18*), *Arabidopsis* (e.g., *SAG12*), Chinese flowering cabbage (e.g., *BrSAG12*), tobacco (e.g., *SAG1*), and wheat (e.g., *TaSAG3*) [104,112,125,143,144]. Conversely, disruption of jasmonate signaling reduces *SAG* expression and delays leaf senescence, as demonstrated in mutants affecting the JA receptor COI1 (*coi1-1* in *Arabidopsis*, *oscoi1b* in rice, and *NtCOI1-RNAi* in tobacco) or in transgenic plants with RNA interference (RNAi)-mediated silencing [98,144,146]. Delays in senescence have also been observed in mutants of key jasmonate signaling components, such as jaz1Δ3a (a JAZ1 mutant), and MYC transcription factor mutants (*myc2*, *myc3*, *myc4*, *myc2/3/4* in *Arabidopsis*, *SlMYC2-RNAi* in tomato), highlighting their critical roles in jasmonate signaling [98,144,146]. Mutants exhibiting delayed senescence, often displaying a stay-green phenotype, may have significant agricultural value by extending photosynthetic activity and potentially increasing crop yields [147].

However, interpreting JA as a primary trigger of senescence is not straightforward and requires distinction between JA accumulation and jasmonate signaling activity. Evidence from drought-related studies indicates that JA can act as a regulatory signal when its accumulation occurs early and is coupled to JA-Ile formation and activation of downstream transcriptional networks, particularly under water deficit conditions [148,149,150]. In contrast, during natural or dark-induced senescence, JA accumulation often coincides with or follows senescence onset and may not activate canonical JA-responsive genes [151,152].

Biochemical evidence further suggests that, under these latter conditions, increased JA levels largely reflect chloroplast membrane lipid degradation and oxylipin metabolism rather than active hormonal signaling [151]. Accordingly, JA accumulation during senescence may represent either a causal regulatory signal or a metabolic consequence of tissue degradation, depending on stress context, developmental stage, and signaling competence.

These context dependencies indicate that framing JA as strictly “pro-senescence” or “anti-senescence” oversimplifies its role. Instead, JA functions as a conditional regulator that calibrates senescence timing according to stress severity, duration, genotype, and tissue type. Under moderate, recoverable drought, jasmonate signaling promotes protective responses that delay terminal senescence [153], whereas under severe or terminal drought, similar pathways can accelerate programmed senescence of expendable tissues to enhance whole-plant survival [154]. Thus, the central challenge is not determining whether JA causes or results from senescence, but identifying the molecular decision points that shift jasmonate signaling from a survival-promoting to a senescence-executing role as stress intensity exceeds recovery thresholds.

JA plays a context-dependent role in leaf senescence. Exogenous MeJA induces *SAGs* in various species, while disruption of jasmonate signaling delays senescence and can produce stay-green phenotypes. However, JA accumulation during natural or dark-induced senescence often reflects tissue degradation rather than active signaling. Under moderate stress, JA can delay senescence by promoting protective responses, whereas severe stress triggers JA-induced programmed senescence. Thus, JA acts as a conditional regulator, with its effects determined by stress, developmental stage, and signaling context.

### 3.6. Genetic and Transcriptomic Evidence for Jasmonate-Induced Senescence Regulation

To gain deeper insight into JA-mediated regulation of leaf senescence, transgenic lines overexpressing specific genes have been generated, exhibiting accelerated senescence phenotypes. For instance, *MdZAT10/STZ-OX* (*Salt Tolerance Zinc Finger*) and *MdABI5-OX* (*Abscisic Acid-Insensitive 5*) lines showed early senescence [132]. Further analyses revealed that MdZAT10 enhances the transcriptional activity of *MdABI5*, thereby promoting senescence. Conversely, the JA-responsive protein MdBT2 interacts directly with MdZAT10, reducing its stability by promoting ubiquitination and degradation, which attenuates MdZAT10 activity and delays MdZAT10-mediated leaf senescence [132]. An and colleagues [125] demonstrated that *MdBBX37-OX* (B-box 37) lines also exhibit accelerated senescence, indicating that MdBBX37 acts as a positive regulator. Their model suggests that MdBBX37 stabilizes MdbHLH93, which enhances transcriptional activation of *MdSAG19*, promoting JA-induced leaf senescence. In the absence of JA, the repressor MdJAZ2 interacts with MdBBX37, weakening its interaction with MdbHLH93 and thereby delaying senescence [125]. Interestingly, not all BBX proteins act as positive regulators; for example, heterologous expression of the chrysanthemum transcription factor *CmBBX22* in *Arabidopsis* delays leaf senescence under ABA treatment by negatively regulating *SAGs* and chlorophyll catabolic genes, including *SAG29*, *NYE1*, *NYE2*, and *NYC1* [128].

Accelerated JA-induced senescence was also observed in the *mta* (*methyltransferase A*) mutant of *Arabidopsis* [112]. MTA, together with Methyltransferase B (MTB) and FKBP Interacting Protein 37 (FIP37), forms the m^6^A writer complex, which catalyzes N^6^-methyladenosine (m^6^A) modification on mRNA, regulating gene expression at the epi-transcriptomic level [155]. The *mta* mutant showed over-accumulation of senescence-related transcripts, including ORE1, SAG21, NAP, and NYE1, leading to accelerated senescence during DILS (Table 1).

Transcriptomic analyses during leaf senescence have revealed that transcription factors from the NAC (20 members) and WRKY (18 members) families constitute the largest groups involved [156]. Functional studies have confirmed their roles in senescence regulation. For example, *wrky57* mutants display accelerated senescence under MeJA treatment, with upregulation of *SAGs*, whereas *WRKY57* overexpression lines (*WRKY57-OX*) exhibit delayed senescence. Chromatin immunoprecipitation (ChIP) experiments revealed that WRKY57 binds promoter regions of *SEN4* and *SAG12*, repressing their transcription (Table 1) [141]. Interestingly, different WRKY family members can exert opposite effects; WRKY42 acts as a positive regulator, as overexpression of wheat *TaWRKY42B* (*TaWRKY42B-OX*) accelerates leaf senescence and upregulates *SAGs*. These lines also show altered expression of JA biosynthesis and signaling genes, while *TaWRKY42B*-silenced lines display delayed senescence, indicating that TaWRKY42B enhances JA levels to accelerate senescence [104].

Recent advances in single-cell RNA sequencing (scRNA-seq) have provided unprecedented resolution to study leaf senescence, revealing cellular heterogeneity and dynamic, cell-type-specific regulatory processes that are obscured in bulk analyses. scRNA-seq in *Arabidopsis* has uncovered tightly coordinated onset and progression of senescence across major cell types. Weighted Gene Co-expression Network Analyses (WGCNA) identified hundreds of hub genes likely acting in concert to orchestrate the senescence program, providing novel insights into the spatial and temporal allocation of carbon and nitrogen and the redistribution of resources from source leaves to sink tissues [157].

Transgenic and mutant studies reveal key regulators of JA-induced leaf senescence. Genes like *MdZAT10*, *MdABI5*, and *MdBBX37* accelerate senescence, while repressors such as MdBT2, MdJAZ2, and some BBX proteins delay it. NAC and WRKY transcription factors further modulate senescence by controlling *SAGs* and JA-related genes. scRNA-seq shows coordinated, cell-type-specific regulation and identifies hub genes governing nutrient redistribution and senescence progression.

**Table 1:** Examples of modulation of gene expression in different plant species during senescence induced by JA/dark or under normal light condition.

Species	Mutant/Transgenic Line/Cultivar	Leaf Senescence Assays	Modulation in Genes Expression Level	Phenotype of Mutant vs. WT Under Specific Treatment or Cultivar Treated/Untreated	References
Apple (*Malus domestica*)	*Arabidopsis* * and apple lines (* indicates the only transgenic *Arabidopsis* line overexpressing an apple gene) *MdZAT10/STZ-OX* (*Salt Tolerance Zinc Finger*), *MdABI5-OX* (*Abscisic Acid Intensive 5*) *, *MdBT2-OX*	DILS+MeJA, detached leaves were floated on 3 mM of MES buffer supplied with 100 µM MeJA kept in the dark for 3 d	upregulated: *NYC1*, *NYE1*	Accelerated senescence (*MdZAT10*-OX, *MdABI5*-OX): decreased total chlorophyll content, decreased F_v_/F_m_; MdABI5 and MdTB2 interact with MdZAT10 (Y2H); Delayed senescence (*MdBT2*-OX)	[132]
	*Arabidopsis* and apple lines, *MdBBX37*-OX (*B Box Protein*), *MdSINA3*-OX (*Seven In Absentia3*), as*MdbHLH93* (antisensce) *MdJAZ2*-OX, *MdBBX37*-OX/*MdJAZ2*-OX	DILS+MeJA, detached leaves were kept in darkness for 15 d (apple) and 3 d (*Arabidopsis*), treatment with ABA, MeJA, ACC	upregulated: *MdSAG18*, *MdNYE1*, *MdNYC1*, *MdORE1*	Accelerated senescence (*MdBBX37*-OX), decreased chlorophyll content, MdBBX37 directly interacts with MdbHLH93 (Y2H) Delayed senescence *asMdbHLH93*	[125]
*Arabidopsis* (*Arabidopsis thaliana*)	*mta* (*methyltransferase a*)	DILS, 3 or 6 d	Changed only after 3 ^/6 * d upregulated: (when is ^ it is change in gene expression only after 3 days, when * only after 6 days, without ^ or * both after 3 and 6 days) *ETR2*, *JAZ10*, *NAP*, *NYE1/*called also SGR *, *ORE1*, *OXI1 ^*, *SAG12*, *SAG13*, *SAG21*, TRX-H5 *, *WRKY6*, *WRKY53* downregulated: CAB1 *, CP33B *, *GLK1/GLK2*, PIF4 *, RBCS1A *	Accelerated senescence: decreased total chlorophyll content (*a*+*b*); increased ion leakage represented as conductivity; photosystem damage decreased F_v_/F_m_, NPQ, RFD_Lss; increased level of JA-Ile (after 6 d treatment), ABA, camalexin (after 6 d treatment)	[112]
	*vir-1* (*virilizer1*)		upregulated: *SAG21*, *SAG113*, *WRKY6*, *WRKY53*	Accelerated senescence	
	*ect2ect4* (*ethylene response 2/4*)		-	Accelerated senescence	
	*coi1-1* (*coronatine insensitive1*)	DILS+MeJA, Detached leaves were floated on 3 mL of water supplied with 100 µM MeJA kept in the dark for 6 d	upregulated: *RBSC*, *CAB* downregulated: *SAG12*, *SAG13*, *SAG29*, *SAG113*, *SEN4*	Delayed senescence, increased chlorophyll content, F_v_/F_m_, decreased ion leakage	[98]
	*jaz1∆3a* (*ja zim-domain1*)		upregulated: *CAB*, *RBSC* downregulated: *SAG12*, *SAG13*, *SAG29*, *SAG113*, *SEN4*	Delayed senescence, increased chlorophyll content, F_v_/F_m_, decreased ion leakage	
	*myc2*, *myc3*, *myc4*, *myc2/3/4*		upregulated: *RBSC*, *CAB* downregulated: *SAG12*, *SAG13*, *SAG29*, *SAG113*, *SEN4*	Delayed senescence, increased chlorophyll content, F_v_/F_m_ (*myc2*, *myc2/3*, *myc2/3/4*), decreased ion leakage (*myc2*, *myc2/3*, *myc2/3/4*).	
	EC (Evening Complex) mutants *elf3* (*early flowering3*), *elf4* (*early flowering4*), *elf3/4*, *lux* (*lux arrhythmo*)	DILS+MeJA, detached leaves were floated on 6 mL of water supplied with 100 µM MeJA kept in the dark for 3–5 d or kept in dark	upregulated: *SAG13*, *SAG29*, *SAG113*, *SEN4* downregulated: *RBCS*	Accelerated senescence, decreased chlorophyll content, increased ion leakage, decreased JA content	[85]
	*wrky57*, *WRKY-OX*	MeJA+light, leaves were floated on water supplemented with 100 µM MeJA, and/or 30 µM IAA and kept in weak light at 22 °C, analysis after 3, 5 d	upregulated in *wkry57*: *SEN4*, *SAG12*, *SAG18*, *SAG20*	Accelerated senescence, decreased chlorophyll content, increased cell death rate; *WRKY*-OX opposite phenotype	[141]
Barley (*Hordeum vulgare* L.)	cv. Golden Promise	DILS, pots with seedlings on seventh day of growth were transferred to dark conditions, analysis on 3, 7, 10 d	The Barley Gene Expression Microarrays (Agilent) upregulated: *NOL*, *RCCR* downregulated: *RUBISCO LS*, *PSBA*, *PSBC* (3, 10 d), *PSBO*, *PSBC*, *LHCB1* (7, 10 d), *LHCB4* (3, 10 d), *LHCB5*, *LHCB6*, *LHCA1*, *PSY*, *PDS*, *LYC* (3, 7 d)	Accelerated senescence, leaves yellowing, necrosis (from 7 d), gradual loss of chlorophyll autofluorescence, changes in chloroplast shape (from lenticular to more spherical, thylakoids had swollen)- 3 d, plastoglobuli appearance- 7d, breakdown of chloroplasts–10 d; structure/shape of the nucleus were accompanied by nuclear DNA fragmentation, nuclei became irregular–7d	[84]
Chinese flowering cabbages (*Brassica rapa*)	cv. Parachinensis	Sprayed with 100 µM MeJA, stored in the incubators at 15 °C and collected after 1, 3, 5, and 7 d	upregulated: *BrSAG12* (without 3 d), *BrSAG19*, *BrPAO* (without 7 d), *BrNYC1*, *BrPPH1*, *BrERF2*, *BrSGR1*, *BrLOX4* (without 2 d), *BrAOC3*, *BrOPR3*	Accelerated senescence, decreased chlorophyll content; photosystem damage: decreased F_v_/F_m_ (3, 5, 7 d), ETR (3, 5, 7 d), increased JA content; transient overexpression of *BrERF72* promotes leaf senescence in tobacco; BrERF72 activates *BrLOX4*, *BrAOC3*, and *BrOPR3* expression (dual-luciferase assays in tobacco leaves)	[143]
Rice (*Oryza sativa*)	cv. Dongjin	Sprayed with 10 mM MeJA, analysis after 6, 30 and 78 h after treatment	upregulated: *SGR*, *RCCR*, *CATa*, *CATb*, *APXa*, *CATb* downregulated: Mg-chelatase subunits (*CHLD*, *CHLH*, *CHLI*), *LHCB1*, *LHCB6*, *PORB*	Accelerated senescence, inhibition of growth of young leaves 30 h after treatment, leaf yellowing after 72 h after treatment; decreased chlorophyll content (30 and 78 h); increased DAB-staining (6, 30 and 78 h); MDA content (78 h); photosystem damage: decreased F_v_/F_m_, ETR, qP, NPQ after 78 h	[99]
	*osdof24-D* (gain-of-function mutant) *OsDOF24-OX*	DILS, detached leaves were floated on 3 mM MES buffer, then incubated at 28 °C in darkness	downregulated: *SGR*, *NYC1*, *NYC3*, *OsI57*, *OsI85*, *OsNAP*, *OsLOX2*, *OsLOX8*, *OsHI-LOX*, *OsAOS1*, *OsAOS2*, *OsMYC2*, *OsCOI1a* (only 4 d)	Delayed senescence, increased chlorophyll content, increased F_v_/F_m_, decreased JA level	[103]
	*oscoi1b* (*coronatine insensitive 1b*)	DILS, detached leaves from 3-week-old plants or the whole plants were incubated in complete darkness, analysis 4 d	vs. WT at 4th day of DILS unchanged: *LOX*, *AOS2* downregulated: *VSP2*, *PDF1.2*, *ORE1.2*, *ORE9*, *SAG12*, *NYC1*, *PAO*, *ABI5*, *EEL*, *EIN3*, *ERF1*, *ICS1*, *S3H*	Delayed senescence, higher chlorophyll content, higher F_v_/F_m_, lower ion leakage	[146]
	*sgr* (*staygreen*) *SGR*-OX	During dark-induced, detached leaf senescence and natural senescence	control vs. dark-induced senescence (*Arabidopsis*) upregulated: *SGN1/2*	*sgr* maintains greenness during leaf senescence, the slower degradation of Chls; *SGR*-OX, yellowish-brown leaves, SGR interacts with LHCPII	[158]
Tobacco (*Nicotiana tabacum* L.)	*NtCOI1*-RNAi	7-week-old plants were daily sprayed with 100 μM MeJA for one or two weeks; DILS, the detached leaf floated in the solution of 100 μM MeJA in darkness or light conditions for 6 d	downregulated both under light and dark conditions in detached leaves: *SAG12*, *SAG-L1*, *SAG21-L* unchanged in whole plant in light condition: *SAG12*, *SAG-L1*, *SAG21-L*	Delayed senescence in whole plants and in detached leaves, increased chlorophyll content under light (whole plant) and light/dark conditions (detached leaves)	[144]
Tomato (*Solanum lycopersicum*)	*SlMYC2-*RNAi	DILS, leaves were floated either on mock solution (control) or 100 μM MeJA, analysis on 2, 4, 6, 8 d	upregulated: *PAO* downregulated: *RCA*, *SBPase*	Accelerated senescence, CO_2_ assimilation rate decreased, chlorophyll content decreased, electrolyte leakage increased; *SLMYC2*-RNAi lower symptoms of senescence vs. WT	[133]
Wheat (*Triticum aestivum* L.)	‘ShiLuan 02–1’ *TaWRKY42-B-OE Arabidopsis* lines *TaWRKY42-B*-silenced *atwrky53*	DILS, leaves were placed into dishes with 10 mL water and were kept in the dark for 5 or 6 days; DILS+MeJA, leaves were treated with 100 μM MeJA solution and were kept in the dark for 4 or 5 days	*TaWRKY4*2-B-silenced lines downregulated *TaSAG3*, *TaSAG5* *TaWRKY42*-B-OX lines upregulated *AtSAG12*, *AtSEN4*, *AtRBCS*, *AtCAB1*, *AtLOX1*, *AtLOX2*, *AtLOX3*, *AtVSP2*, *AtMYC2*, *AtMYC3*, *AtMYC4*	*TaWRKY4*2-B-silenced lines: Delayed senescence, lower chlorophyll degradation and ion leakage rate, decreased DAB staining; after dark treatment the stay-green phenotype was observed *TaWRKY42*-B-OX lines: Accelerated senescence *atwrky53* Delayed senescence	[104]

### 3.7. Experimental Context and Translational Limitations of DILS-Derived Mechanisms

DILS is a widely used experimental model because it triggers senescence processes quickly and reproducibly. However, it differs fundamentally from chronic drought experienced by field-grown plants. In DILS, senescence is primarily caused by sudden loss of photosynthetic carbon and acute energy starvation. In contrast, field drought develops progressively over weeks under continued photoperiodic cycling, allowing acclimation responses such as osmotic adjustment, metabolic reprogramming, and root–shoot coordination [159,160]. Consequently, DILS typically produces rapid, synchronized chlorophyll loss, while drought-associated senescence is gradual, spatially heterogeneous, and strongly dependent on genotype and environment [84,161]. It is also very important to consider leaf senescence in field-grown plants as a key adaptive mechanism to drought stress, which may be crucial for the survival of many plant species. Drought-induced senescence promotes the redistribution of nutrients from older leaves to actively growing organs, such as young leaves, flowers, or developing fruits, thereby enabling their continued growth under stress conditions. Moreover, drought-induced leaf senescence, especially when followed by leaf abscission, helps to reduce transpirational water loss and thus supports the maintenance of overall plant water balance [161].

Hormonal and signaling contexts also diverge markedly between DILS and field drought. Field drought senescence is embedded in complex multi-hormone networks dominated by abscisic acid and integrated with jasmonates, ethylene, cytokinins, and often salicylic acid [162]. In contrast, DILS imposes extreme carbon starvation and overrides normal circadian and light-dependent regulatory inputs, potentially activating signaling routes that are not dominant under drought [84,159]. This distinction is particularly relevant for jasmonates, as jasmonate accumulation in DILS frequently reflects JA-Ile–dependent signaling [24,148], whereas drought-associated oxylipin responses may differ in timing, composition, and signaling competence [46].

Importantly, DILS and field drought differ not only in stress kinetics and physiological context, but also in the predominant functional modes of JA accumulation and signaling. In DILS, JA accumulation often occurs under abrupt carbon starvation and altered circadian regulation, conditions that can favor starvation- or damage-associated oxylipin production and, in some cases, limited engagement of canonical COI1-dependent transcriptional responses. By contrast, under progressive field drought, jasmonates typically operate within an ABA-centered hormonal network and may involve OPDA-dependent and partially COI1-independent signaling branches. These differences constrain the direct extrapolation of JA-associated regulatory logic derived from DILS to chronic drought-induced senescence in field-grown plants.

Despite these limitations, DILS remains useful for dissecting conserved downstream senescence processes, including chlorophyll catabolism and core senescence-associated gene networks. However, upstream regulatory mechanisms, hormonal hierarchy, and temporal dynamics observed in DILS should be regarded as hypothesis-generating and require validation under progressive drought and field-relevant conditions before agronomic extrapolation.

### 3.8. ABA–Jasmonate Interactions During Drought-Induced Leaf Senescence

Drought-induced leaf senescence is governed by complex hormonal networks in which ABA and jasmonates play central but non-equivalent roles. While both hormones accumulate during water deficit and influence senescence-related processes, accumulating genetic, molecular, and physiological evidence indicates that their interaction is best described as context-dependent integration with functional hierarchy, rather than as a simple linear signaling cascade.

Several studies report rapid accumulation of jasmonates during the early phases of drought stress (within hours of stress imposition), in some cases preceding detectable increases in ABA [163,164]. These observations support a role for jasmonates and related oxylipins as early stress-responsive signals, potentially contributing to initial perception of and local adjustment to dehydration. However, as drought progresses from acute to sustained water deficit, ABA accumulation becomes dominant and more persistent. ABA biosynthesis is tightly coupled to osmotic stress perception and is rapidly amplified through transcriptional induction of key biosynthetic enzymes, enabling systemic signaling and long-term physiological reprogramming [162]. Importantly, the timing and magnitude of ABA accumulation correlate more consistently with senescence progression than jasmonate levels, particularly under progressive, field-relevant drought conditions [165].

Genetic evidence strongly supports a central and non-redundant role for ABA signaling in drought-induced leaf senescence. Disruption of core ABA signaling components, particularly the sucrose non-fermenting 1-related protein kinase 2 (SnRK2), abolishes ABA-induced senescence responses, demonstrating that ABA signaling is necessary for senescence commitment under drought [162,165]. Conversely, enhancement of ABA perception or signaling capacity is sufficient to accelerate drought-induced senescence, even in the absence of direct manipulation of jasmonate pathways [162,165]. At the molecular level, ABA promotes drought-induced senescence through pyrabactin resistance 9 (PYL9)/ABA receptor protein-protein phosphatase 2C-sucrose nonfermenting 1-related protein kinase 2s (PYL9–PP2C–SnRK2) signaling module, which activates downstream transcription factors such as ABA-responsive element-binding factors (ABFs) and related to ABA-insensitive 3/VP1 transcription factor (RAV1). These, in turn, induce senescence-induced NAC transcription factors, including ORE1 and AtNAP, leading to activation of *SAGs* and execution of the senescence program. The ABA receptor PYL9 promotes ABA-induced leaf senescence in both *Arabidopsis* and rice, whereas PYL8 regulates dark-induced leaf senescence in *Arabidopsis* [162,166]. PYL8-overexpressing plants exhibited enhanced leaf yellowing, increased membrane ion leakage, reduced chlorophyll content during dark-induced senescence, and elevated expression of *SAGs* [166]. This ABA-driven regulatory axis operates independently of jasmonate perception, underscoring ABA’s hierarchical dominance during sustained drought stress [162,164,165]. However, transcriptomic analyses show that ABA-responsive regulators such as ABA-insensitive growth 1 (ABIG1) can modulate expression of JA-pathway genes (e.g., *ORA59*, *TIFY* family), and more recent work points to synergistic ABA–JA interactions in drought tolerance and senescence, suggesting non-trivial crosstalk rather than strict pathway independence [167].

Jasmonate signaling components, particularly the COI1–JAZ–MYC module, interact with ABA signaling at multiple molecular nodes [168,169]. Degradation of JAZ repressors can enhance the activity of ABA-responsive transcription factors such as ABI5, thereby potentiating ABA-induced gene expression [169]. Interestingly, ABI5 was shown to play a role in ABA-dependent regulation of barley responses to drought stress at both the seedling and heading stages [170,171]. The basic helix–loop–helix transcription factor MYC2 emerges as a key point of convergence between the two pathways. MYC2 is transcriptionally responsive to ABA, participates in ABA-dependent stress gene regulation, and simultaneously controls jasmonate biosynthesis and signaling outputs [172,173]. A direct interaction between the ABA receptor PYL6 and MYC2 has been demonstrated and proposed as a putative link between ABA and jasmonate signaling pathways [172]. Through this dual role, MYC2 enables feedforward amplification loops in which ABA induces jasmonate production, and jasmonates in turn enhance ABA responsiveness. Such integration allows fine-tuning of senescence progression according to stress severity, duration, and developmental context [172,173].

Notably, ABA can also repress specific branches of jasmonate signaling through ABA-induced transcription factors [172,174] further emphasizing the asymmetrical nature of their interaction. This capacity for selective reinforcement or attenuation of jasmonate outputs enables ABA to coordinate senescence with other drought-adaptive processes, including stomatal regulation and antioxidant defense [149,173]. Representative examples of stress-induced changes in gene expression linked to premature leaf senescence across different plant species are shown in Table 2.

Collectively, the evidence reviewed above is consistent with a hierarchical but dynamically integrated model of ABA-jasmonate interaction during drought-induced leaf senescence. In this framework, ABA functions as the primary integrative signal that determines whether and when senescence is initiated in response to declining plant water status. Jasmonates do not generally override this ABA-driven decision but instead act as context-dependent modulators that refine the timing, spatial patterning, and physiological execution of senescence, including effects on stomatal behavior, antioxidant capacity, and stress-responsive transcription. This division of regulatory labor reconciles reports of early jasmonate accumulation during drought with the consistent requirement for intact ABA signaling to achieve irreversible senescence commitment. Thus, in most studied systems, drought-induced leaf senescence emerges as an ABA-centered process whose outcomes are fine-tuned by jasmonate signaling rather than controlled by it. Thus, drought-induced leaf senescence is the outcome of hierarchically organized yet dynamically integrated hormonal signaling, with ABA acting as the dominant regulator and jasmonates serving context-dependent modulatory roles.

**Table 2:** Examples of modulation of gene expression in different plant species under stress-induced premature leaf senescence.

Species	Mutant/Transgenic Line/Cultivar	Stress Treatment	Modulation in Genes Expression Level	Phenotype	References
*Arabidopsis* (*Arabidopsis thaliana*)	*ANAC092*-IOE; (ANAC092 is also called AtNAC2 and ORE1; Inducible OX)	Seedlings were grown hydroponically in control condition or in 150 mM NaCl for 4 d. Leaves detached from 25-day-old wild-type (WT) and *anac092-1* plants were incubated for 4 days in water (control) or 150 mM NaCl.	Microarray-based expression profiling experiment, *ANAC92*-IOE, 170 genes were up-regulated, among them 78 were senescence-associated genes, and among them 24 were up-regulated by salinity	Delayed leaf senescence of the *anac092-1* mutant, which retained higher chlorophyll levels under stress than the WT.	[175,176]
Grapes (*Vitis vinifera* L.)	‘Muscat Hamburg’, *VvSGR*-OX in *Arabidopsis*	Grapes were grown in plastic pots in optimal water condition till the seedlings reached 120 cm in length. The drought treatment was applied by withholding watering for 12 d and soil water content (SWC) was measured.	RNA-Seq study, WT: drought vs. control upregulated: *SGR*, *NYC1*, *PAO*, *bZIP40/ABF2*, *WRKY54/75/70*, *ANAC019*, *MYC2*, *NAC0002*, *NAC019*, *NAC048* downregulated: *GAI*, *GAI1*, *AHK4*, *AHK2*, *RR22*, *RR9-1*, *RR9-2*, *RR6*, *RR4* *VvSGR*-OX in *Arabidopsis vs. WT* upregulated: *AtCAO*, *AtCLH1/2*, *AtHCAR*, *AtPPH*, *AtNOL AtNYC1*, *AtSAG12*, *AtSGR1*	Drought led to accelerated senescence, increased MDA and H_2_O_2_ levels, decreased CAT activity, Chl level, Pn, Tr, gs, Ci; *VvSGR*-OX lines in *Arabodopsis* show early yellowing, lower Chl level.	[134]
Pepper (*Capsicum annuum* L.)	‘B12’, *CaPAO*-silenced plant (VIGS). *CaPAO*-OX in tobacco	Leaf senescence was induced by floating disk leaves in various concentrations (0, 300, 400 and 500 mM) of NaCl solution with continuous lighting at 25 °C for 3 days. Salt treatment was applied to six-leaf-stage plants uprooted from the soil, and their roots were soaked in 400 mM NaCl. Plant leaves were sprayed with either 0.57 mM ABA, 5 mM SA or 1 M MeJA. Analysis after 2, 4, 8, 12, 24 and 48 h.	control vs. treatment (hormone, stress) upregulated: *CaPAO*	*CaPAO*-silenced lines show delayed salt-induced leaf senescence (stay-green phenotype). *CaPAO*-OX in tobacco accelerated the process of salt-induced leaf senescence, decreased chlorophyll content and increased MDA level.	[177]
Wheat (*Triticum aestivum* L.)	Fu287 (F287), drought-sensitive; Shannong20 (SN20), drought-resistant	Drought induced the flag leaf senescence at the grain-filling stage. Plants were grown in cement pools, density 270 plants/m^2^, ECH2O soil moisture monitoring systems were used (Decagon, San Francisco, CA, USA).	SN20 vs. Fu287 under drought upregulation: *Cu/Zn-SOD*, *Mn-SOD*, *Fe-SOD*, *POD*, *CAT*, *APX*	SN20 vs. Fu287 under drought showed higher thousand-grain weight, lower damage to the chloroplast membrane system during leaf senescence, delayed flag leaf senescence based on SPAD value (r_0,_ r_max_, r_aver,_ Chl_total_, Chl_per_,M); SA, JA, Z, ZR, GA_3_ higher content.	[178]

## 4. Conclusions and Future Perspectives

### 4.1. Summary of Current Knowledge

Jasmonates, including JA and MeJA, are central regulators of plant growth, development, and stress responses. Their bioactive form, JA-Ile, initiates signal transduction through the COI1-JAZ-MYC module, controlling the expression of genes involved in growth–defense trade-offs. Beyond canonical jasmonate signaling, OPDA serves as an independent signaling molecule, modulating stress responses and development in a COI1-independent manner. Leaf senescence, a genetically programmed process influenced by both endogenous cues and environmental stresses, is tightly regulated by jasmonate signaling. JA promotes senescence through activation of *SAGs*, modulation of transcription factors such as MYC, WRKY, and NAC families, and coordination with other phytohormones. Chlorophyll degradation and chloroplast disassembly, accompanied by transcriptomic and epi-transcriptomic reprogramming, are key physiological hallmarks of senescence. Recent studies using transgenic lines, mutants, and scRNA-seq have revealed the complex regulatory networks controlling JA-mediated leaf senescence, highlighting the interplay between transcriptional, post-transcriptional, and epigenetic mechanisms. In several senescence contexts, particularly during natural aging or dark-induced leaf senescence, elevated JA levels can largely reflect oxylipin production associated with chloroplast membrane turnover, rather than a direct regulatory role in senescence initiation. In contrast, under abiotic stress conditions such as drought and salinity, jasmonate signaling can function as a context-dependent regulatory module whose effects on senescence progression depend on timing, tissue competence, and interaction with upstream hormonal pathways, most notably ABA.

### 4.2. Gaps in Understanding and Future Research Directions

Despite significant progress, several critical gaps remain. First, the precise mechanisms underlying OPDA-specific signaling and its integration with JA-dependent pathways are not fully understood. Second, the spatiotemporal dynamics of JA and OPDA signaling in different cell types and tissues during senescence require further exploration. The role of epi-transcriptomic modifications, including m^6^A methylation, in fine-tuning senescence-related gene expression remains an emerging field that warrants deeper investigation. Additionally, the functional diversification within TF families, such as WRKY, NAC, and BBX, and their cross-talk with other phytohormonal pathways need to be elucidated at both cellular and tissue levels. Advanced tools, such as scRNA-seq, spatial transcriptomics, and genome-editing technologies, offer opportunities to address these gaps and reveal regulatory nodes amenable to manipulation.

A key conceptual gap is the frequent conflation of jasmonate accumulation with jasmonate signaling competence. Elevated JA levels during senescence are often interpreted as regulatory activity, although multiple studies indicate that JA can accumulate without proportionate activation of COI1-dependent transcription, particularly in dark-induced and age-dependent senescence. Distinguishing signaling-active jasmonates (such as JA–Ile) from oxylipin by-products of membrane turnover therefore remains a major unresolved experimental and conceptual challenge.

Moreover, mechanistic insights derived from DILS and acute hormone treatments remain insufficiently validated under progressive, field-relevant drought and salinity conditions. Differences in hormonal hierarchy, temporal dynamics, and tissue-specific responses under chronic stress limit direct agronomic extrapolation.

Finally, although ABA is widely regarded as the principal regulator of drought-induced senescence regulation, the quantitative and temporal rules governing ABA–jasmonate interactions, particularly the thresholds that shift jasmonate function from stress adaptation to senescence execution, remain poorly defined. Resolving these questions will require integrative, time-resolved and tissue-specific approaches under realistic stress regimes.

### 4.3. Potential Applications in Crop Improvement

Understanding jasmonate-mediated leaf senescence has direct implications for agriculture. Modulating jasmonate signaling or its downstream regulators can be exploited to delay premature senescence, extend photosynthetic activity, and enhance biomass and yield in crops. The stay-green phenotype, resulting from targeted manipulation of JAZ repressors, MYC transcription factors, or BBX proteins, represents a promising strategy to improve crop performance under environmental stresses, including drought, salinity, and heat. Moreover, fine-tuning OPDA and jasmonate signaling pathways could optimize stress resilience without compromising growth.

In this context, effective crop improvement strategies will require targeting jasmonate signaling competence (e.g., JA–Ile perception and COI1–JAZ–TF modules) and downstream regulatory decision nodes, rather than simply altering bulk jasmonate levels. Integration of molecular insights with breeding and biotechnological approaches, such as CRISPR/Cas-mediated genome editing or transgenic overexpression of key regulatory genes, offers potential for developing next-generation crops with improved productivity, nutrient use efficiency, and post-harvest quality.

In conclusion, jasmonate signaling constitutes a central regulatory hub linking stress responses, developmental processes, and leaf senescence. Continued research into its molecular mechanisms and cross-talk with other pathways will not only deepen our understanding of plant biology but also provide actionable strategies for sustainable crop improvement in the face of climate change.

## Data Availability

No new data were created or analyzed in this study. Data sharing is not applicable to this article.

## Abbreviations

The following abbreviations are used in this manuscript:

α-LeA	α-Linolenic Acid
ABA	Abscisic Acid
ABF	ABA-Responsive Element-Binding Factors
ABI5	ABA Insensitive 5
ABIG1	ABA-Insensitive Growth 1
ACC	1-Aminocyclopropane-1carboxylic Acid/Ethylene Precursor
AOC	Allene Oxide Cyclase
AOS	Allene Oxide Synthase
AUXs	Auxins
APX	Ascorbate Peroxidase
BRs	Brassinosteroids
CAB1	Chlorophyll A/B Binding Protein1
CAT	Catalase
CHL	Chlorophyll
ChIP	Chromatin immunoprecipitation
*cis*-(+)-OPDA	*cis*-(+)-12-Oxophytodienoic Acid
CKS	Cytokinins
CLH1/2	Chlorophyllase
COI1	Coronatine-Insensitive 1
CTS	Comatose
CP33B	PSBA RNA-Binding Protein Chloroplast Ribonucleoprotein 33b
CYP20-3	Cyclophilin 20-3
CYS	Cysteine
DAB	3,3′-Diaminobenzidine
DILS	Dark-Induced Leaf Senescence
DIS	Dark Induced Senescence
EEL	Enhanced Em Level
ERF3/72	Ethylene Response Factor 3/72
ETR	Electron Transport Rates
ETR2	Ethylene Response 2
EIN3/EIL	Ethylene Insensitive 3
ET	Ethylene
FIP37	FKBP Interacting Protein 37
F_v_/F_m_	Maximum Quantum Efficiency Of PSII
GAs	Gibberellins
GLK1/GLK2	Golden 2-Like Transcription Factor 1/2
GSH	Glutathione
HCAR	7-Hydroxymethyl Chlorophyll *a* Reductase
13-HPOT	(13S)-Hydroperoxyoctadecatrienoic Acid
IAA	Indole-3-Acetic Acid
I57	3-Ketoacyl-Coa Thiolase
I85	Isocitrate Lyase
ICS1	Isochorismate Synthase 1
JA	Jasmonic Acid
JA-ILE	Jasmonic Acid–Isoleucine Conjugate
JAT1/2	Jasmonate Transporter
JAR1	Jasmonoyl Isoleucine Conjugate Synthase 1
JASSY	Jasmonate-Associated Secretory System
JAZ	Jasmonate ZIM-Domain Protein
LHCA1	PSI Light-Harvesting Complex Gene1
LHCB1/4/5/6	Light-Harvesting Complex of PSII Subunit 1/4/5/6
13-LOX	13-Lipoxygenase
LYC	Lycopene Cyclase
MDA	Malondialdehyde
MED25	Mediator Complex Subunit 25
MeJA	Methyl Jasmonate
MES	2-(N-Morpholino)ethanesulfonic acid
MTA	Methyltransferase A
MTB	Methyltransferase B
MYC2	BHLH ZIP Transcription Factor MYC2
NAP	NAC-Like, Activated By AP3/PI
NINJA	Novel INteractor of JAZ
NOL	NYC1-Like Chlorophyll *b* Reductase
NPQ	Non- Photochemical Quenching
NYC1	Non-Yellow Coloring 1
NYE1	Nonyellowing 1/SGR1, Staygreen
OASTL-B	O-acetylserine(thiol)lyase B
OPC-8	3-Oxo-2-(2-Pentenyl)-Cyclopentane-1-Octanoic Acid
OPDA	12-Oxo-Phytodienoic Acid
OPDAT1	OPDA Transporter 1
OPR3	OPDA Reductase 3
ORE1	Oresara1
OX	Lines With Overexpression Of Targeted Gene
OXI1	Oxidative Signal Inducible1
PAGs	Photosynthesis-Associated Genes
PAO1	Pheophorbide A Oxygenase1
PDF1	Plant Defensin 1.2
PDS	Phytoene Desaturase
PIF4	Phytochrome-Interacting Factor 4
PLA1	Phospholipase A1
POD	Peroxidase
POL II	RNA Polymerase II
PORB	Pchlide Oxidoreductase B
PPH1	Pheophytin Pheophorbide Hydrolase1
PP2C	Protein Phosphatase 2C
PSBA/C	PSII Reaction Center Protein A/C
PSBP	PSII Subunit P
PSBO	PSII Manganese-Stabilizing Protein
PSY	Phytoene Synthase
PYL9	Pyrabactin Resistance 9
qP	Photochemical Quenching
RAV1	ABA-Insensitive 3/VP1 Transcription Factor
RBCS	Ribulose Bisphosphate Carboxylase Small Chain
RC	PSII Reaction Centers
RCA	Rubisco Activase
RCCR	Red Chlorophyll Catabolite Reductase
RFD_LSS	Relative Fluorescence Decline
RNAi	RNA Interference
ROS	Reactive Oxygen Species
SA	Salicylic Acid
SAGs	Senescence Associated Genes
SAT1	Serine Acetyltransferase 1
SBPase	Sedoheptulose-1,7-Bisphosphatase
SCF	SKP1–Cullin–F-Box Ubiquitin E3 Ligase Complex
scRNA-seq	single-cell RNA sequencing
SEN4	Senescence 4
SLS	Strigolactones
SnRK2	Sucrose Non-Fermenting 1-Related Protein Kinase 2
SOD	Superoxide Dismutase
S3H	Salicylic Acid 3 Hydroxylase
TFS	Transcription Factors
TGA	TGACG-Binding Transcription Factors
TPL	Topless
TRX-H5	Thioredoxin H5
UB	Ubiquitin
VSP2	Vegetative Storage Protein 2
WT	Wild-Type
WGCNA	Weighted Gene Co-expression Network Analyses
Y2H	Yeast Two-Hybrid Assay
